# Impact of cardiosphere-derived cells on the maladapted right ventricular muscle in a rat sugen/hypoxia model of pulmonary hypertension with right ventricular dysfunction

**DOI:** 10.1371/journal.pone.0321895

**Published:** 2025-05-12

**Authors:** Ryan C. Middleton, Oleg A. Karpov, Mario Fournier, Simion Kreimer, Mitra Mastali, Weixin Liu, Liang Li, Norbert F. Voelkel, Jennifer E. Van Eyk, Eduardo Marbán, Michael I. Lewis

**Affiliations:** 1 Smidt Heart Institute, Cedars-Sinai Medical Center, Los Angeles, California, United States of America; 2 Amsterdam University Medical Centers, Amsterdam, Netherlands; 3 Division of Pulmonary/Critical Care, Cedars-Sinai Medical Center, Los Angeles, California, United States of America.; Ohio State University, UNITED STATES OF AMERICA

## Abstract

**Background and aims:**

With pulmonary arterial hypertension (PAH), right ventricular (RV) function is a major determinant of survival. Despite current therapies, maladaptive changes ensue in the RV muscle of PAH patients, culminating in RV dysfunction and failure. The aims of the study were to evaluate the impact of intra-coronary (IC) cardiosphere-derived cells (CDCs) in attenuating the maladaptive pathobiology in the RV muscle and evaluating mechanisms underlying improvements in RV function.

**Methods:**

Two groups of the Sugen/Hypoxia rat model of PAH, exhibiting significantly reduced RV function, via TAPSE measurements, received either intracoronary infusion of CDCs or PBS placebo. Immunohistochemistry methods were used to assess RV pathobiological changes. Additionally, advanced proteomics were employed to examine protein signaling pathways and upstream regulators.

**Results:**

RV muscle capillarity was significantly reduced in the PAH rats while RV muscle fibrosis was increased. IC CDCs significantly increased RV muscle capillarity back to levels noted in healthy rats and reduced RV free wall fibrosis. Further, a significant reduction in iNOS^+^ (M1) macrophages was also observed within the RV free wall in CDC-treated animals. Proteomic analysis of RV muscle in CDC- or PBS-treated PAH rats showed alterations in protein pathways related to inflammation, fibrosis, autophagy, cell vitality, and angiogenesis. These changes were consistent with putative coordination by a small number of key upstream regulators (MYC, TP53, HNF4A, TGFB1, and KRAS). TAPSE was significantly reduced in PBS-treated animals but was maintained at or above baseline levels in CDC-treated animals.

**Conclusions:**

CDC therapy can significantly impact the maladaptive milieu of the RV myocardium in advanced PAH, by altering several pathobiological pathways. Such adjunctive therapy, in addition to those employed to reduce pulmonary vascular resistance, would be a great advance in managing RV failure, for which no effective current approved therapies exist.

## Introduction

Pulmonary Arterial Hypertension (PAH) is a progressive condition for which there is no cure and survival is poor, even in the modern treatment era [1]. PAH is characterized by remodeling of the pulmonary arteries and arterioles (narrowing +/- obstruction), resulting in elevated pulmonary artery pressure, increased right ventricular (RV) afterload, and the subsequent development of right heart dysfunction and right heart failure (RHF). Most patients do not succumb to high, sustained, pulmonary arterial pressures, but rather RV dysfunction leading RHF [2].

With PAH, the RV initially adjusts to the high afterload presented to it by adaptive and physiologic hypertrophy. This lowers RV wall stress (Law of Laplace) and preserves or improves RV function, often for prolonged periods. However, for unknown reasons, decompensation and RHF ensue [3], characterized by eccentric hypertrophy and RV dilatation, with a fall in cardiac output, impaired contractile reserve, further reduction in exercise capacity and RV diastolic dysfunction [2–8]. Patients with PAH commonly develop RV dysfunction and failure despite being treated with the maximum combination of PAH-specific therapies. While prostanoids and phosphodiesterase-5 inhibitors can exert positive influences on the RV [9], this is not sustained. No current approved therapies have shown efficacy in reversing, or even slowing, the progression of RHF.

Maladaptive alterations in the dysfunctional or failed RV muscle have been well characterized in animal models [2,6,8,10]. These include increased inflammation [11, 12] and RV fibrosis [3], impaired angiogenesis and reduced capillary density, that induces ischemia and contributes to a hibernating myocardium [13–15], cardiomyocyte apoptosis [8,16], oxidative stress [8], impaired efferocytosis [11, 12] and altered metabolism with a shift to uncoupled glycolysis [6,17].

Cardiosphere-derived cells (CDCs) are heart-derived, stromal/progenitor cells that exhibit a variety of salutary properties on injured tissue that are mediated by the release of exosomes and their payload of bioactive factors, such as non-coding RNAs [18–21]. First reported in 2007 [22], CDCs are multipotent and clonogenic [19,23]. CDCs exhibit potent angiogenic [24–26], anti-fibrotic [26–28], anti-inflammatory [25,29–31], immune modulating [32, 33], and anti-apoptotic properties [20,24,29]. They can attenuate both oxidative and nitrosative stress [29], as well as attract endogenous stem cells to sites of vascular injury [34]. Of note, intracoronary (IC) administration of CDCs reversed pathophysiologic properties in a rat model of heart failure with preserved ejection fraction (HFpEF), including reducing myocardial fibrosis (LV and RV), inflammation and cytokine/chemokine expression, while improving capillary density [25]. The use of CDCs is potentially attractive as they have been proven safe in hundreds of human infusions, with evidence of disease-modifying bioactivity in various fibro-inflammatory conditions. The therapeutic effects of CDCs are currently being evaluated in a phase three clinical trial for Duchenne muscular dystrophy (DMD) and its associated cardiomyopathy.

The major objective of this study was to evaluate the impact of IC CDCs on the pathobiological changes observed in the maladapted RV muscle in the Sugen/Hypoxia rat model of PAH exhibiting RV dysfunction (as defined by a tricuspid annular systolic plane excursion (TAPSE)). To this end, we employed histological, immunohistochemistry and advanced discovery proteomic analyses on the RV myocardium in healthy control rats and PAH rats receiving either IC-CDCs or PBS placebo. Our data show that CDCs restored a more “adaptive” RV milieu and unique proteomic changes across several domains that are likely driving these results, as well as the demonstration of key upstream regulators.

## Methods

### Animal care and anesthesia

Male Sprague-Dawley rats were housed in pairs with a 12-hour light/dark cycle at ambient temperature and fed Purina rat chow and water *ad libitum*. Prior to all surgical procedures and echocardiography measurements, animals were initially anesthetized with 5% Isoflurane for approximately 3 minutes within a gas anesthesia induction chamber. Foot pad compression was used to confirm unconsciousness. Then, animals were placed upon a heated stage and 2% isofluorane was administered via nose cone throughout all TAPSE measurements and surgical procedures. After each procedure, animals were monitored closely by research staff until consciousness was regained. All experimental protocols were approved by the Cedars-Sinai Animal Care and Use Committee (IACUC).

### Pain management and euthanasia

Animals were assessed twice daily for signs of pain or stress, including inactivity, changes in breathing, loss of appetite, piloerection, red material around eyes or nose, and other signs indicative of stress or pain. No signs of pain were observed after surgery. If signs of pain had been observed, the animals would have received 0.05 mg/kg buprenorphine, and the Cedars-Sinai veterinary staff would have been consulted. For euthanasia purposes, rodents were placed under deep general anesthetized (5% isofluorane for 5 minutes within a gas anesthesia induction chamber) and unconsciousness was confirmed by several toepad compressions. Upon confirmation, animals would then be subjected to exsanguination via heart removal.

### Sugen/hypoxia model

All experimental protocols were approved by the Cedars-Sinai Animal Care and Use Committee (IACUC). 8-week-old, 200g male Sprague Dawley rats were given subcutaneous injections (20 mg/kg) of Sugen (SU5416, Sigma) [35]. The SU5416 was suspended in vehicle consisting of 0.5% carboxymethylcellulose sodium, 0.9% sodium chloride, 0.4% polysorbate 80, and 0.9% benzyl alcohol in deionized water. Control animals received vehicle only. Rats were housed in the hypoxia chamber (Biospherix) for four weeks, at 10% oxygen using nitrogen gas displacement, with daily monitoring. At the end of four weeks, the rats were returned to ambient oxygen levels for a minimum of five days before any procedures requiring anesthesia were conducted. We did not observe any premature animal deaths during or after Sugen/Hypoxia induction, however we did observe that animals placed under anesthesia, before the five-day waiting period had ended, showed a reduction in heart rate and body temperature, and the procedure was suspended.

The Sugen/Hypoxia model of PAH generates different sex-specific phenotypes. Whereas male rats develop advanced right heart failure, the female rats develop very limited right heart failure in this model [36]. Therefore, only male rats were used in this study.

See online supplement for further animal care details and sex selection rationale.

### CDC generation, animal selection, intracoronary (IC) CDC delivery, echocardiography-derived TAPSE and immunohistochemistry

Sprague-Dawley rat CDCs were generated from strain-matched hearts as described previously [37]. Following five days post hypoxia, only rats with TAPSE measurements on echocardiography of less than 2.7mm were used in the study; a value > 2SD below normal values in healthy rats as previously determined by our group and others. This inclusion threshold is common for 300g PAH Sprague-Dawley rats [38–40]. The animals were injected with either 500,000 cells in 100μL of sterile PBS or PBS-only control into the left ventricle following temporary aortic occlusion [31] to achieve IC delivery. (See justification supporting this technique in the supplement and from our prior studies [31,41,42], outlining both imaging/technical aspects and functional data). Four weeks after CDC delivery, RV pressure was measured hemodynamically, and then the heart was removed for histology. The fixed hearts were sectioned and stained using standard procedures. See online method supplement for further details, including CDC tracing, RV immunohistochemistry and TAPSE measurements.

### Animal groups

Three groups were studied. 1) PAH rats given IC CDCs (n=15), 2) PAH rats given IC phosphate-buffered saline (PBS; n=13) and 3) healthy, age-matched rats (n=7).

### Proteomic analysis

Frozen RV free wall was solubilized in 2% SDS. Proteins were precipitated with acetone, and reduced, alkylated and digested with Trypsin/Lys-C prior to liquid chromatography-mass spectrometry (LC-MS) analysis (Orbitrap Elite mass spectrometer). MS spectra were searched against the Swiss-Prot reviewed rat and mouse FASTA database [43, 44] with target-decoy modelling using Peptide Prophet [45] on Scaffold 3 version 1.4.1 (Proteome Software,) with quantification via Skyline software [46]. Biological and functional process identification was performed using Fundamental Enrichment analysis software and integrated and visualized using PINE [47]*. See the online supplement for more details.*

### Statistical analysis

Statistical analysis was performed to compare differences between independent groups using one-way ANOVA. Post-hoc analysis (Tukey’s multiple comparisons test) was used to compare differences between independent groups, if a significant interaction was found. An alpha level of 0.05 was used to compare differences between groups, and overall significance. Significant difference between the indicated groups (brackets) are represented by * (p<0.05), ** (p<0.01), *** (p<0.001), and **** (p<0.0001. Values are expressed as means ± SEM. Researchers performing the analysis were blinded to the animal treatment groups.

## Results

### General proteomic data

From the LC-MS based analyses carried out on healthy, PAH-PBS, and PAH-CDC rodent RV tissue homogenates, 1513 unambiguous proteins were identified (S1 Table**).** These protein expression values were compared to quantify differentially expressed proteins (DEPs, i.e., proteins significantly increased or decreased when comparing conditions). In total, 271 significant DEPs were identified from RV isolated from PAH animals infused with PBS, with approximately the same number of significant DEPs (278 protein IDs) in CDC-treated animals (S2 and S3 Tables).

**Fig 1:**
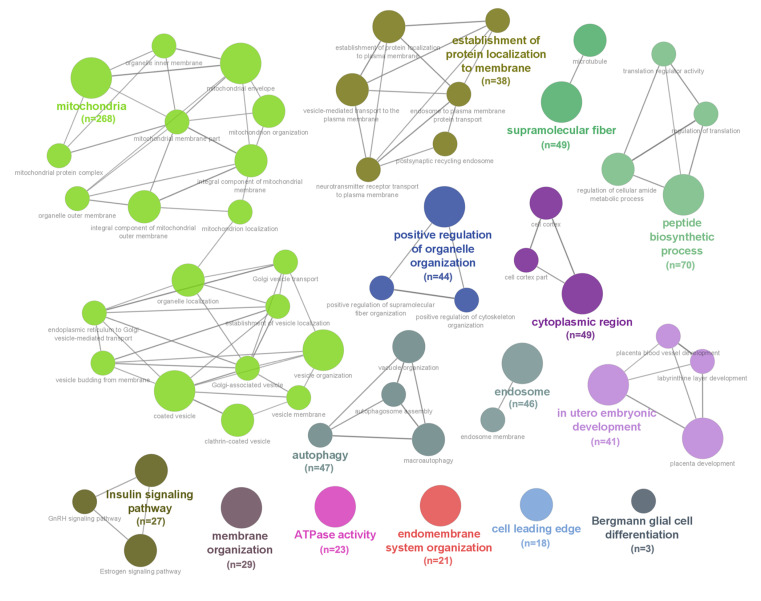
The functional network of proteins that differed significantly between CDC-treated and PBS-treated PAH animals.

To identify potential underlying mechanisms driving the proteomic changes and link them to the demonstrated phenotypes, DEPs were screened for their known subcellular localizations (sub-proteomes), gene ontologies, cellular functions, associated cellular pathways, and known upstream regulators. The cell functions associated with the largest number of DEPs in CDC-treated PAH animals compared to PBS-infused controls were “mitochondrial and cellular transport” (268 protein IDs), followed by “protein translation” (70 protein IDs), “regulation and/or part of the sarcomeric myofilament/cytoskeleton” (49 protein IDs), and “autophagy” (47 protein IDs) (Fig 1, S1 and S2 Fig**).** Taken together, the protein changes in the RV muscle indicate that CDC-treatment of PAH reflects a broad impact on inflammation, fibrosis, autophagy, and contractile units. CDC-specific DEPs were found to be involved in fibrosis, hypertrophy, vascularity, and immune cell infiltration (S4 Table).

Network diagram representing the 278 significantly differentiated proteins (18.3% of total proteins identified) identified using mass spectrometry-based proteomic analysis between CDC-treated and PBS-treated PAH animals. Gene ontologies of various proteins are highlighted in different colors, with relative sizes of the network bubbles relating to the number of proteins per ontology listed under each network node.

### Pathobiology and proteomic linkage

#### CDC-induced increase in vascularity.

**Fig 2:**
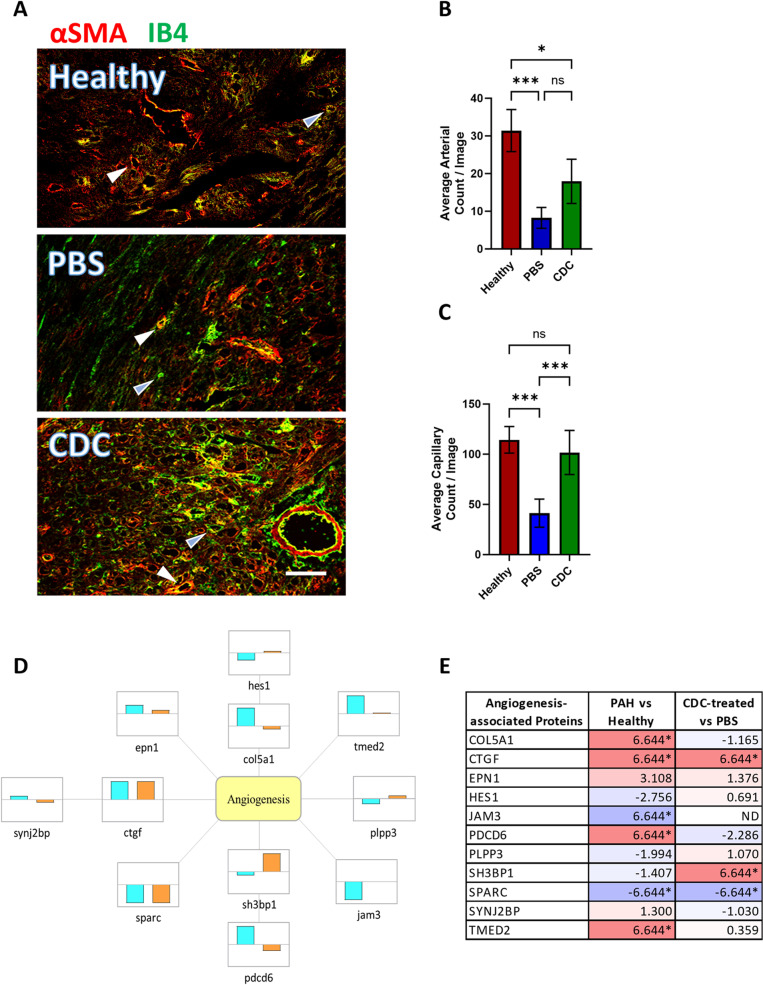
RV free wall vascularity assessment in PAH rats at 4 weeks post treatment. (A) Representative images of capillaries (Isolectin B4, green) and arterioles (Isolectin B4 and smooth muscle actin, green and red) within the RV free wall from healthy, PBS-treated and CDC-treated PAH animals are shown. Examples of capillaries and arterioles are indicated by blue and white arrowheads, respectively. Analysis of the mean number of (B) arterioles and (C) capillaries per image (± SEM) are shown. Significant difference between the indicated groups (brackets) are represented by * (p<0.05), ** (p<0.01), ***(p<0.001). Note that some of the extracellular green signal observed in the PAH-PBS and PAH-CDC images may be from fibrosis autofluorescence. Scale bar = 50µm. (D) Angiogenesis-associated proteins that are differentially expressed in Healthy vs. PAH-PBS animals and PAH-CDC vs PAH-PBS animals are shown (cyan and orange bars, respectively), with bar direction indicating either up or down regulation. (E) Table listing the fold changes (log_2_(FC)) of proteins for the relevant samples. Darker purple colors (- numbers) represent downregulation and lighter pink colors (+ numbers) represent up-regulation of proteins. Values denoted by * represent proteins that were identified only in certain samples (only in CDC-treated or only in control sample), therefore may not be indicative of true changes.

Assessment of vascularity by immunohistochemistry revealed that RV muscle capillarity was reduced by ~ 60% in PAH rats receiving PBS while those in whom CDCs were administered had significantly higher numbers of capillaries, that closely approached healthy rat RV muscle values (Fig 2A and B). Further, there was a trend toward increased arterioles in the RV free wall in CDC-treated rats compared to the PBS rats (p=0.052). Twelve CDC-specific DEPs known to modulate angiogenesis were significantly changed, and importantly, the expression of 8 DEPs reverted towards healthy levels following CDC-treatment. These included collagen alpha 1 (V) chain (COL5A1) and other ECM regulatory proteins (*e.g.,* Transcription factor, HES-1, regulatory proteins junctional adhesion molecule 3 (JAM3), and SH3 domain-binding protein 1 (SH3 BP1).While tissue analysis cannot unambiguously assign observed changes to specific cell types, cardiomyocytes and smooth muscle cells are certainly altered with CDC treatment, given the changes in cardiac-specific myosin light chain 4 (MYL4) and smooth muscle cell-derived Calmodulin-1 (CAM) and calponin-3 (CNN3).

#### CDC-induced alteration in cardiac fibrosis.

**Fig 3:**
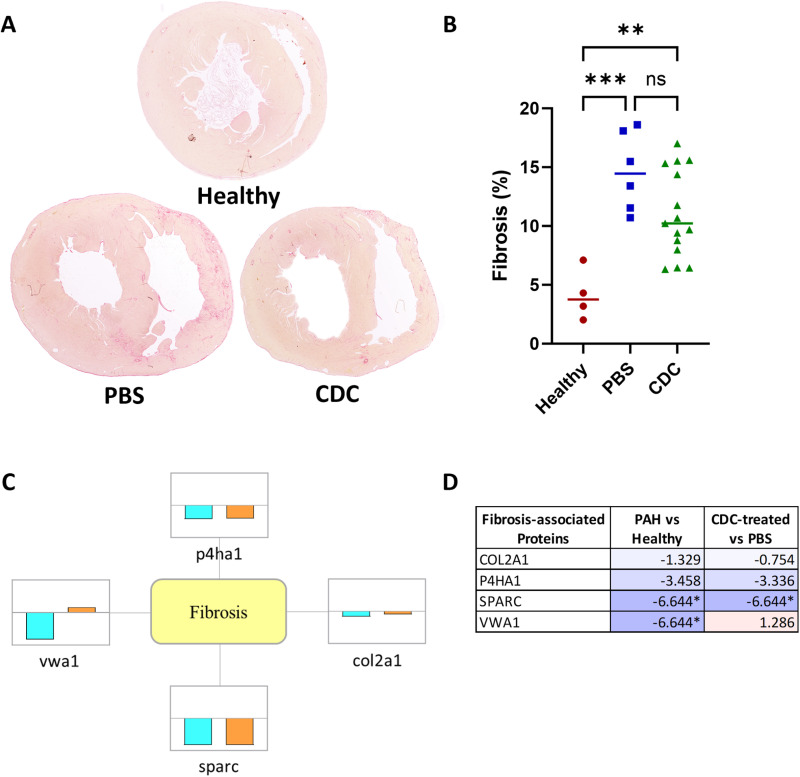
Histological and proteomic fibrosis assessment within the RV free wall at four weeks post treatment. (A) Transverse cryosections of hearts from the three animal groups, stained with Picrosirius red to show the amount and locations of fibrosis, are shown. (B) RV free wall fibrosis (represented as a percentage of total RV tissue area) is shown for all animals, along with their mean values (horizontal bar) ± SEM. Significant difference between the indicated groups (brackets) are represented by * (p<0.05), ** (p<0.01), ***(p<0.001). (C) Fibrosis-associated, differential protein expression is represented by individual boxes with the cyan (PAH-PBS vs Healthy) and orange (PAH-CDC vs. PAH-PBS) boxes visually indicating up or down regulation across a central horizontal axis. (D) Table lists the fold changes (log_2_(FC)) of proteins for the relevant samples. Darker purple colors (- numbers) represent downregulation and lighter pink colors (+ numbers) represent up-regulation of proteins. Values denoted by * represent proteins that were identified only in certain samples (only in CDC-treated or only in control sample), therefore may not be indicative of true changes.

The RV free wall of CDC-treated rats showed a trend (p=0.098) of reduced collagen staining (11.2% of total tissue), compared to PBS (14.6%) and healthy rats (4.1%) (Fig 3A and B). Specific to CDC treatment, Collagen alpha-1 (II) chain (COL2A1) was reduced (while other collagens were not), as were several regulatory proteins involved in the extracellular matrix remodeling [*e.g.,* Prolyl 4-hyhroxylase subunit alpha-1 (P4HA1) and secreted protein acidic and rich in cysteine (SPARC) were decreased, while von Willebrand factor A domain-containing protein 1 (VWA1) was increased] (Fig 3C and D).

#### Altered immune cell Infiltration due to CDC treatment.

**Fig 4:**
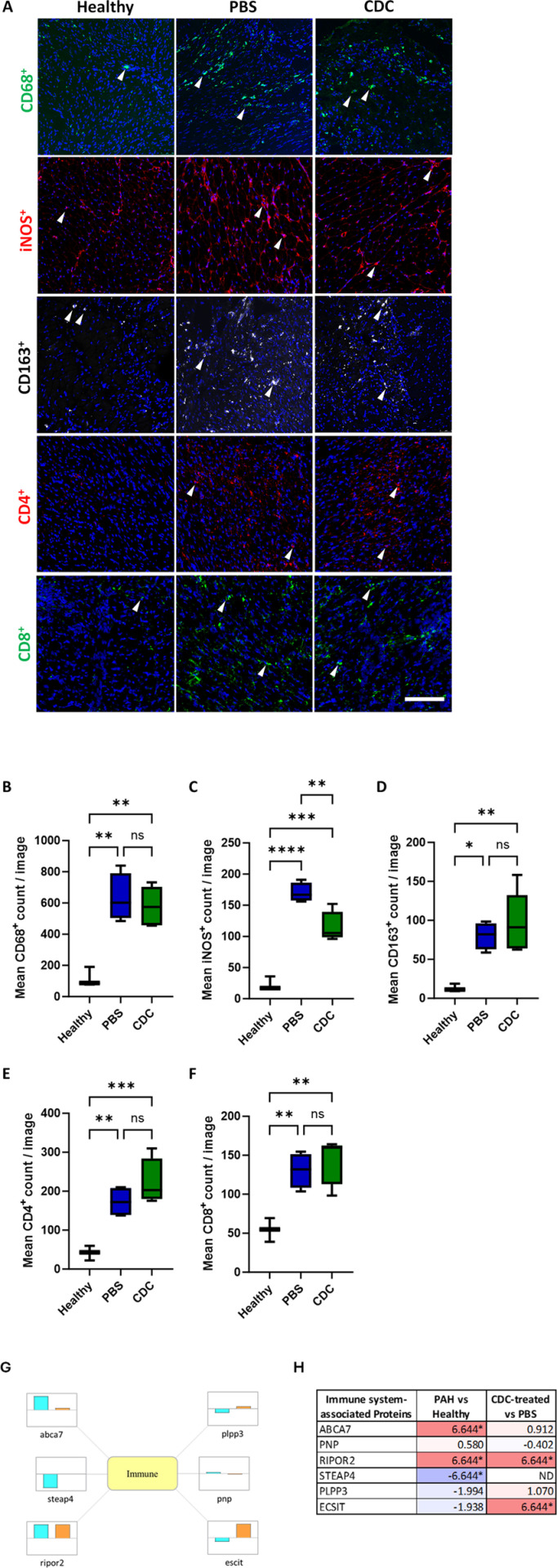
Perivascular inflammation assessment in healthy, CDC- and PBS-treated PAH rat RV free wall. Immunohistochemical analysis of RV free wall tissue sections from healthy, CDC-treated and PBS-treated PAH animals at 4 weeks post treatment are shown. (A) Panel of representative images taken from the three experimental groups for macrophage markers (top row: CD68^+^, second row: iNOS^+^, third row: CD163^+^) and T-cell markers (fourth row: CD4^+^, fifth row: CD8^+^). The small, white arrowheads indicate examples of the cells that were counted within each image). Graphical representations of the mean immune cell count per image (± SEM) are shown for (B) CD68^+^, (C) iNOS^+^, (D) CD163^+^, (E) CD4^+^, and (F) CD8^+^, for each of the three treatment groups. Significant differences between the indicated groups (brackets) are represented by * (p<0.05), ** (p<0.01), ***(p<0.001). Scale bar = 200µm. (G) Immune system-associated differential protein expression is represented by individual boxes with the cyan (PAH-PBS vs Healthy) and orange (PAH-CDC vs. PAH-PBS) bars visually indicating up or down regulation across a central horizontal axis. (H) Table lists the fold changes (log2(FC)) of proteins for the relevant samples. Darker purple colors (- numbers) represent downregulation and lighter pink colors (+ numbers) represent up-regulation of proteins. Values denoted by * represent proteins that were identified only in certain samples (only in CDC-treated or only in control sample), therefore may not be indicative of true changes.

As the anti-inflammatory properties of CDC therapy have been demonstrated in several studies [19,25,31,33,48–51], leukocyte populations within the RV free wall were assessed. A significant increase in the CD68^+^ macrophages within the RV free wall was observed in PAH animals compared to healthy animals, however no significant difference in total macrophage number between CDC-treated and PBS-treated PAH animals was found (Fig 4A and B). When observing macrophage subsets, iNOS^+^ macrophages (typically indicative of a pro-inflammatory M1 phenotype) were significantly lower in CDC-treated animals compared to PBS-treated control (Fig 4A and C), while CD163^+^ (pro-regenerative M2 phenotype) macrophages were comparable in the two groups (Fig 4A and D). Additionally, CD4^+^ (i.e., helper) T-cells, while not statistically significant (p=0.230), trended toward higher levels in CDC-treated animals compared to PBS-treated animals (Fig 4A and E). No changes in CD8^+^ (i.e., cytotoxic) T-cell number were observed between the two PAH groups (Fig 4A and F). Staining controls can be found in the supplement (S3 Fig).

Several DEPs involved in immune signaling and inflammation were altered in CDC-treated PAH versus PBS-treated PAH rats. CDC treatment led to increased phagocytosis signaling, and reduced secretion of inflammatory cytokines associated with DEPs: ATP-binding cassette sub-family A member 7 (ABCA7), Purine nucleoside phosphorylase (PNP), and Rho family-interacting cell polarization regulator 2 (RIPOR2), amongst others (Fig 4G and H).

#### Cardiomyocyte hypertrophy.

**Fig 5:**
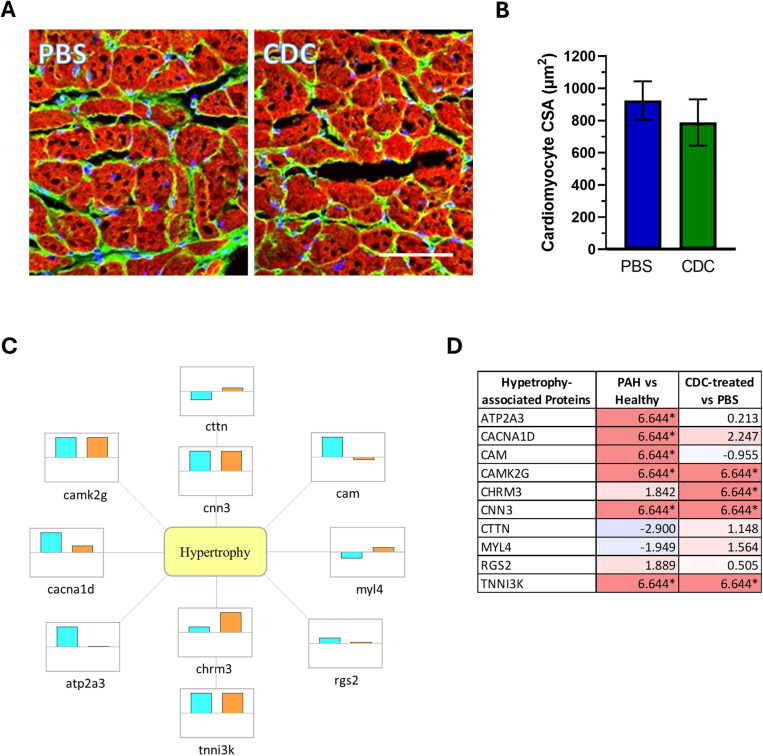
Cardiomyocyte hypertrophy assessment of the rodent RV free wall at 4 weeks post treatment. (A) Representative images of cardiomyocyte cross-sections within the RV free wall of PBS- or CDC-treated PAH animals are shown. The cell membranes are shown in green, and the myofibrils are shown in red. Scale bar = 100um. (B) Analysis of the mean cross-sectional areas of cardiomyocytes (± SEM) from the different PAH treatment groups are shown. (C) Hypertrophy-associated protein expression is represented by individual boxes with the cyan (PAH-PBS vs. Healthy) and orange (PAH-CDC vs. PAH-PBS) bars visually indicating up or down regulation across a central horizontal axis. (D) Table lists the fold changes (log2(FC)) of proteins for the relevant samples. Darker purple colors (- numbers) represent downregulation and lighter pink colors (+ numbers) represent up-regulation of proteins. Values denoted by * represent proteins that were identified only in certain samples (only in CDC-treated or only in control sample), therefore may not be indicative of true changes.

Microscopic analysis of cardiomyocyte size revealed similar cross-sectional areas (CSAs) in CDC-treated animals compared to PBS control (Fig 5A and B). These PAH rat cardiomyocyte CSA values are remarkably similar to those measured in age and strain matched, Sugen/hypoxia-induced PAH rat studies [52, 53]. At a proteomic level however, CDCs did impact on some proteins that could promote cardiomyocyte hypertrophy and/or contractility, such as cholinergic receptor muscarinic 3 (CHRM3) and troponin 1 interacting kinase (TNNI3K), as well as changes in Ca^2+^-handling and sarcomeric proteins and associated pathways (Fig 5; S4 Table), including calcium voltage -gated channel subunit alpha 1D (CACNA1D) CHRM3, ATPase sarcoplasmic calcium transporting 3 (ATP2A3), and other regulators of G-protein signaling like regulator of G protein signaling 2 (RGS2) and TNNI3K (S4 Table). Of note, MYL4, a cardiac-specific myosin light chain isoform, was upregulated in CDC-treated samples [54, 55].

#### Proteomic analysis of other areas associated with PAH pathobiology.

Additional cell signaling pathways were altered in PAH animals treated with CDC compared to PBS-treated animals. The differential expression of many proteins associated with cell vitality and proliferation, autophagy, metabolism and ROS handling were observed. **(See Supplemental results and** S1 and S2 Figs **for additional information)***.*

#### Predicted upstream regulators for PAH and CDC-responsive proteome changes.

**Fig 6:**
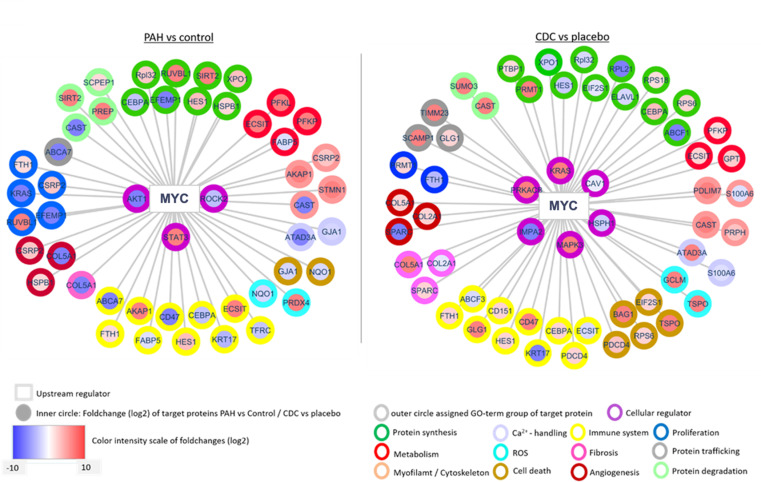
Association of the upstream protein regulator, MYC, with the RV DEPs between CDC and PBS-treated animals.

A bioinformatics search for upstream intracellular signaling effectors identified Myc proto-oncogene protein (MYC), Kirsten rat sarcoma virus (KRAS), Hepatocyte nuclear factor 4-alpha (HNF4A), Cellular tumor antigen p53 (TP53), and Transforming growth factor beta-1 (TGFB1) as potential orchestrators of CDC responses (S5 Table). All of the upstream regulators impact immunoregulatory/inflammatory signaling, with MYC having the largest number of CDC-responsive DEPs involved (Fig 6). MYC itself has 3 downstream regulators (MAP kinase 9 (MAPK9), heat shock protein family 1(HSPH1) and programmed cell death protein 4 (PDCD4)), that are central to inflammatory and fibrotic regulation [56–60]. KRAS is involved in cell proliferation, angiogenesis, and inflammation; TP53 regulates protein expression, immunoregulation, and metabolism; HNF4A regulates protein expression, protein transport, and cell proliferation (S5 Table**).** TGFB1, in addition to influencing immunoregulatory and inflammatory signaling, is also involved in altering sarcomeric proteins and cell proliferation (S5 Table). Taken together, upstream regulator MYC, KRAS, HNF4A, TP53, and TGFB1 seem to have central roles in mediating CDC-induced signaling. These regulators govern transcriptional and translational protein machinery, and protein trafficking, which ultimately drive the salutary changes in CDC-treated animals.

The upstream regulator MYC was identified to link to many of the protein changes (p<0.05) identified in the comparison between the various treatment groups (PAH-PBS vs Healthy (left panel) and PAH-CDC vs PAH-PBS (right panel)). For the inner circle, the color intensity of each protein represents the fold difference (red indicates increases while blue is decreased protein levels). For the outer circle, the specific color represents the gene ontology and biological pathway assigned to the protein.

#### Physiological characteristics.

**Fig 7:**
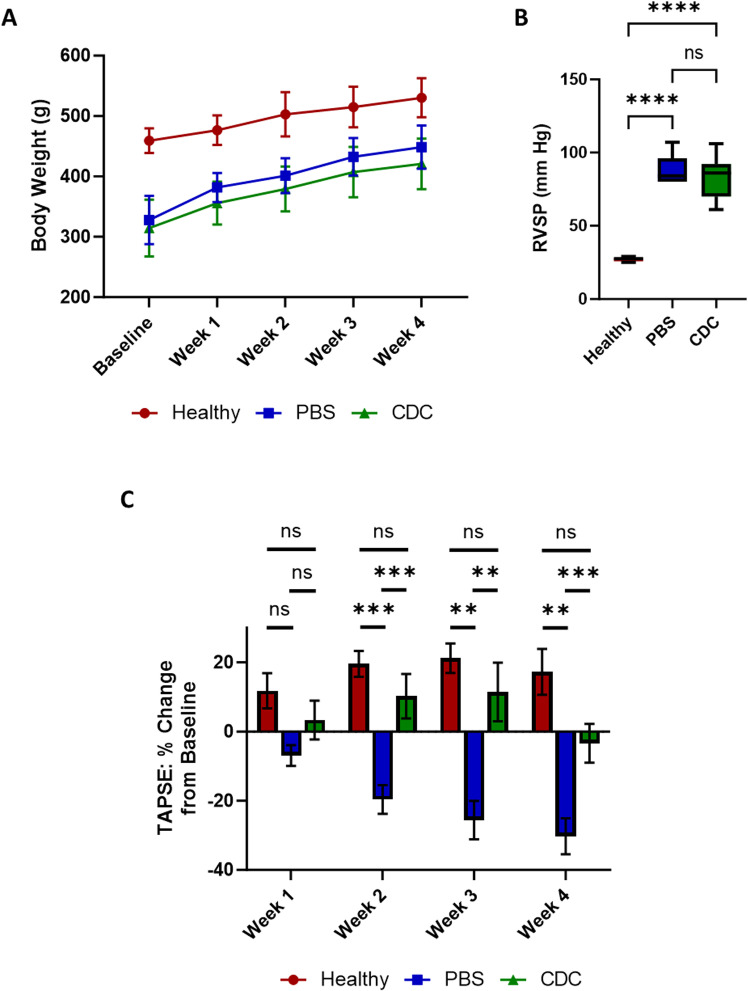
Analysis of rodent body weight, RV chamber pressure, and RV function. (A) Healthy and PAH rat body weight measurements from immediate post-hypoxia (baseline) until 4 weeks post CDC or PBS delivery. (B) RV systolic chamber pressure, measured at 4 weeks post therapeutic delivery, is shown in healthy, CDC-treated and PBS-treated animals. (C) Weekly echocardiography assessments of TAPSE for the three animal groups are shown. TAPSE measurements are displayed as the mean percent change from baseline ± SEM. One-way Anova analysis with multiple comparisons was conducted between the three rodent groups, at each timepoint. Significant difference between the indicated groups (brackets or lines) are represented by * (p<0.05), ** (p<0.01), and *** (p<0.001).

Body weight measurements taken at baseline showed reduced mass in PAH animals compared to healthy animals. As the study progressed, all three groups gained weight at similar rates (Fig 7A). As TAPSE was used to screen the Sugen/Hypoxia rats for inclusion in the study, we continued to follow this marker of RV systolic function. TAPSE measurements taken at 2-, 3-, and 4-weeks post IC CDCs showed significant improvements in RV function compared to PBS-treated animals (Fig 7C, S4 Fig**).** Across the course of the study, TAPSE in CDC-treated animals increased above base line, but not significantly, while the TAPSE in PBS-treated animals significantly decreased below baseline at 3- and 4- weeks (p-values = 0.029 and 0.004, respectively). RV systolic pressures were comparable in CDC- and PBS-treated animals (CDC: 83.55mm Hg; PBS: 88.0mm Hg), both of which were significantly higher than in healthy rats (27.2mm Hg) (Fig 7B**).**

## Discussion

In the Sugen/Hypoxia rat model of severe PAH with impaired RV systolic function, CDCs significantly ameliorated pathobiological derangements in the maladapted RV muscle. This was reflected by an increase in RV free wall capillarity toward levels seen in healthy rats, a decrease in RV muscle fibrosis, and a decrease in pro-inflammatory macrophages. Proteomic analyses provided further mechanistic insights into DEPs and to key upstream regulators impacted by the CDCs to address the pathobiological RV muscle milieu caused by PAH and RV dysfunction. The marker used to screen for RV systolic dysfunction, TAPSE, was maintained above baseline levels in PAH rats receiving CDCs until four weeks post infusion, while the TAPSE for PAH-PBS animals continually decreased over time.

### The maladapted RV muscle milieu with PAH and clinical implications

PAH is a progressive condition for which there is no cure. Even with substantial pharmacologic advances in the modern treatment era, survival remains unacceptably poor [61–63]. Most patients die from RHF, initiated by RV dysfunction [64]*.*

In the maladapted RV muscle, several biochemical and molecular mechanisms have been identified [6,8,10,64]. These include impaired angiogenesis resulting in capillary rarefaction, [8] myocardial ischemia, and an inability to meet the high oxygen demands imposed, [13,15] leading to a “hibernating state”. Inhibition of hypoxia-inducible factor-1α (HIF-1α) by mitochondria-generated reactive oxygen species (ROS) is thought to be a prominent factor [8], as well as epigenetic influence on angiogenesis, such as downregulation of miR126 [14]. Altered RV fibrosis, which is traditionally considered a hallmark of maladaptive RV hypertrophy [2], is likely to drive the progression of both systolic and diastolic dysfunction and failure [65, 66]. Increased oxidative stress contributes to ROS-mediated damage and apoptosis of cardiomyocytes, leading to increased cardiac fibrosis [8]. Fatty acid oxidation normally provides the greatest source of energy (ATP) [6]*.* With the onset of RV dysfunction, however, a metabolic shift to less energy-efficient glycolysis ensues [6], and is associated with an increase in lactate, also referred to as the Warburg phenomenon [17]*.* Inflammation is an important driver of PAH. These effects are described within the RV in animal models [11, 12] and clinically in patients with PAH, such as scleroderma-associated PAH. Cellular mechanisms that drive these changes include cellular apoptosis and impaired cellular autophagy. Apoptosis of RV cardiomyocytes occurs with RHF and is well described in the Sugen/Hypoxia rat model of PAH-associated RHF [8,16].

### Intracoronary administration of CDCs

Our group has previously evaluated the retention of CDCs in the myocardium in the rat following intracoronary administration using the transient aortic occlusion technique following injection of CDCs into the LV [41,67]. Intracoronary delivery was far more effective, uniform and had the highest retention rates in the RV compared to the intraventricular septum and LV delivery. RV myocardial uptake with the same intracoronary delivery paradigm described above has also been reported for mesenchymal stem cells [42]. See the supplement for further information on CDC tracing, RV coronary blood supply contributions, and prior positive functional results using the intracoronary CDC administration technique [25].

### Influences of CDCs on the pathobiological maladapted RV muscle

CDCs can induce multiple positive influences *simultaneously,* to affect several mechanisms proposed to lead to RV dysfunction and failure.

#### RV vascularity.

PAH rats revealed a dramatic reduction in capillarity and small vessels in the RV muscle. Previous studies in PAH have demonstrated that a loss of vasculature was due to reduced expression of angiogenesis factors such as VEGF and angiopoietin 1 [13,15]. Epigenetic factors within the PAH RV muscle, such as reduced expression of Mir-126, which suppresses expression of VEGF inhibitors [14], may also play a role. This reduced vascularity was rescued by CDC treatment with markedly reduced capillarity in PAH-placebo animals returned to levels seen in healthy rats following CDC administration.

RV proteomic analysis in CDC-treated animals found altered expression in several signaling pathways affecting angiogenesis. Angiogenesis-related genes with significant regulation changes related to CDC treatment include SH3 BP1, PDCD6, and CTGF. SH3 BP1, a Rho GTPase that plays a role in angiogenesis and micro-vessel formation as part of the semaphorin-plexin signaling pathway [68] and promotes VEGF secretion [69]. Restoration of gene expression levels was observed in CDC-treated PAH rats. Another regulator of VEGF signaling, and cell apoptosis, Programmed Cell Death 6 (PDCD6) is significantly upregulated in PAH rat RVs but suppressed in CDC-treated animals. PDCD6 has been shown to inhibit angiogenesis through the inhibition of PI3K signaling via VEGR2 interaction [70].

#### RV fibrosis.

Animals that received CDCs showed a trend of decreased RV muscle fibrosis observed within the rodent PAH model described above. Key proteins involved in collagen synthesis and processing, P4HA1 and SPARC, were decreased in CDC-treated animals, as was COL2A1. These proteins are governed by the TGFβ signaling family and are well-known contributors to fibrosis in acute cardiomyopathy and heart failure [4,7,8]. vWA1, an extracellular matrix protein found within cardiomyocyte basement membrane was down-regulated in PAH rodents but demonstrated increased expression in CDC-treated PAH animals. Higher vWA1 expression has been associated with cardiac remodeling [71], and promoting repair, instead of fibrosis, following myocardial infarction injury [72].

#### RV inflammation.

RV tissue immunohistochemistry identified significantly increased numbers of CD68^+^ macrophages in all PAH animals. CDC treatment did not significantly alter that total number of macrophages compared to placebo. However, iNOS-expressing, M1 pro-inflammatory macrophage levels were significantly reduced in CDC-treated rats. Assessment of CD163-expressing, M2 (anti-inflammatory) macrophages were not significantly different between PAH treatment groups. Altered T-cell levels and impaired regulatory T-cell function are also characteristics of PAH [73]. We observed dramatically increased CD4^+^ and CD8^+^ T-cell levels in the RVs of both PAH groups compared to those in the healthy animals. Although not significant, we observed an increasing trend in the presence of CD4^+^ helper T-cells in the RVs of CDC-treated animals, which also include TREGs. This increase may reflect an increase in the subpopulation of TREGs within the CD4^+^ group that are typically suppressed or dysfunctional in PAH. Furthermore, we did not observe significant changes in cytotoxic CD8^+^ T-cells between the PAH treatment groups. The limited inflammatory response observed following CDC treatment may be due to the 1-month time frame that progressed following CDC delivery. CDCs remain within the vasculature for approximately three days following jugular vein infusion, however the therapeutic effects have been measured to last significantly longer [48, 49]. Three- and seven-day time points post-CDC infusion in models of myocardial infarction showed significant reductions in macrophages, T-cells, and other immune infiltrating cells as well as circulating cytokines [33,50].

Proteomic analysis found a limited but specific response in the inflammatory response of CDC-treated PAH animals which included altered macrophage phagocytosis and suppression of inflammatory cytokine secretion. For example, the gene expression of RIPOR2, an inhibitor of leukocyte polarization and migration, was significantly increased in PAH animals, and further increased following CDC treatment. Analysis of peripheral blood cell samples collected from patients with systemic sclerosis–associated PAH demonstrated revealed the upregulation of RIPOR2 expression [74]. Although a decrease in decrease in T-cell presence was not observed in CDC-treated animals, the increase in RIPOR2 may have served a more global function related to inflammation and fibrosis in the RVs of these animals. ECSIT (likely ortholog of mouse signaling intermediate in Toll) is another inflammation-associated gene that demonstrated increased levels of expression following CDC treatment. In macrophages, ECSIT functions as an adapter protein that localizes to the mitochondria, following the activation of toll-like receptors, and promotes ROS production to digest phagocytized microbes [75]. Human cardiac tissue samples taken from patients undergoing cardiac surgery demonstrated a strong, inverse correlation between ECSIT expression and left ventricle hypertrophy and collagen I presence, suggesting that ECSIT plays a cardio-protective role [76]. PAH animals showed a marked decrease in ECSIT that was rescued following CDC infusion, and likely contributed to the increased cardiac function and reduced fibrosis observed in this study.

#### Muscle hypertrophy and contractility.

Although no statistical difference in muscle fiber size was observed, several proteins involved in hypertrophy and contractility were altered in CDC-treated animals. In total, 36 sarcomeric proteins were changed following CDC therapy, of which six are known to regulate contractility. MYL4 is a cardiac gene whose expression is predominantly in the atria in healthy hearts but is overexpressed in the ventricles of failing hearts and is commonly found in hypertrophic cardiomyopathy, dilated cardiomyopathy, and ischemic heart disease [55]. Although the mechanism for regulating MYL4 presence in failing hearts is not fully understood, its increased expression in diseased hearts may be part of a compensatory mechanism for failing hearts to maintain functional output as MYL4 interacts through cAMP-associated PI3K activation to promote cardiomyocyte function and survival [77]. Further, transgenic animal studies that overexpress MYL4 in the left ventricle found that fibers expressing high MYL4 levels exhibited higher maximal velocity and rate of fiber shortening [78] and MYL4 overexpression attenuated heart failure in pressure-overloaded animals [79, 80]. MYL4 expression was significantly higher in CDC-treated PAH animals compared to PBS-treated control and may have contributed to the enhanced function observed via TAPSE by preserving cardiomyocyte viability and enhancing contractility, but without driving further muscle fiber hypertrophy. Additionally, a number of calcium handling genes were up-regulated in the PAH RV following CDC treatment and may further contribute to enhanced function. Interestingly, proteomic analysis also revealed the overexpression of cardiomyocytes hypertrophy regulators with opposing functions, following CDC treatment. CHRM3, a muscarinic acetylcholine receptor associated with smooth muscle function, is overexpressed in hypertrophic hearts and functions to suppress angiotensin II-related hypertrophic signaling [81]. TNNI3K has been shown in several *in vitro* and transgenic animal studies to promote adaptive and maladaptive cardiac hypertrophy by increasing cardiomyocyte area and sarcomere organization [82]. Both proteins are upregulated in the PAH rat RV compared to healthy controls, and both are further upregulated in animals that received CDC therapy, suggesting that conflicting actions on cardiomyocyte hypertrophy are regulating RV muscle fiber growth. Thus, complex interactions are in play with maladapted RV hypertrophy.

#### Upstream regulators and drivers of proteomic changes.

There were several upstream signaling regulators that were associated with protein changes identified following CDC therapy. The proto-oncogene MYC is well described for its role in maintaining cardiac muscle growth, regulates immune cell activation and modulates fibrosis. Most gene changes identified within the MYC signaling pathway have been downregulated in failing right hearts. CDC therapy has been shown to reverse these effects in numerous hypertrophy-associated genes together with suppression of fibrosis-related genes, such as COL2A1. Similarly, the TGFβ superfamily signaling pathway associated genes, are downregulated in failing right hearts, but were rescued in animals receiving CDCs. Another upstream regulator strongly affected by CDC therapy in the RV free wall is the oncogene KRAS, which is involved in the signaling pathways of several cellular responses, such as VEGF-A and SDF-1 expression. KRAS was significantly reduced in failing PAH hearts but its expression was rescued following CDC therapy. Additionally, other upstream signaling factors including HNF4 and TP53, which modulate protein expression, cell transport and immune modulation, had similar expression patterns in failing hearts and were also rescued by CDC therapy.

#### RV systolic function.

A ≥2 SD reduction in TAPSE in PAH rats compared to healthy animals was required for entry into the study. We followed this parameter for four weeks in healthy, and CDC- and PBS-treated PAH rats. TAPSEs showed significant improvements in CDC-treated animals compared to placebo at 2-, 3-, and 4-weeks post-infusion. As the focus of this study was to evaluate the impact of CDCs on the aberrant pathobiological features of the maladapted RV myocardium, we did not explore any additional functional studies, which will be the focus of a separate study on functional RV changes.

## Conclusions and clinical implications

This study showed that CDC administration to the RV myocardium rescued or significantly reversed key pathobiologic abnormalities in the RV muscle with PAH (such as reduced capillarity, inflammation and fibrosis, as well as improved RV function). Further, advanced proteomics provided other signaling pathway insights into RV dysfunction and improvements with CDCs, including key upstream regulators. This could promote future targeted approaches to improve RV function which would be part of the future envisaged precision medicine approach to disorders. Currently however, our data are highly important, as to date we have no approved therapies clinically, that can impact the maladapted RV muscle to improve function and prevent the ensuing RV morbidity and/or mortality in patients with PAH.

Thus, the clinical implications of this study are significant. Current therapeutic approaches are merely supportive (e.g., diuretics etc.) and many patients fail to qualify for bilateral lung transplantation (or heart-lung transplant) because of associated co-morbidities. We envisage repeated IV infusions of CDCs as being an effective therapy, as observed in Phase 2 clinical trials in which Duchenne Muscular Dystrophy patients that received repeated CDC infusions demonstrated impressive improvements in cardiac function and limb muscle strength [83]. This would enable both direct and indirect approaches to treating PAH patients with RV dysfunction using cell therapies. The direct approach would be CDCs and their products entering the coronary circulation from IV infusion to effect RV muscle changes. The indirect approach would be CDCs acting on remodeled pulmonary vasculature to significantly reduce RV afterload and improve RV function via mechanisms related to RV muscle plasticity. This approach to manage a major unmet need in the treatment of patients with advanced PAH complicated by RV dysfunction and failure would be a major potential new therapeutic advance.

### Permission information

The authors do hereby declare that all illustrations and figures in the manuscript are entirely original and do not require reprint permission.

## Supporting information

S1 Table LC-MS analysis identification of unambiguous proteins from RV free wall tissue lysates from healthy, PBS- or CDC-treated PAH rats. (XLSX)

S2 Table Comparison of differentially expressed proteins and associated values between healthy and PAH rodent RV free wall myocardium. (XLSX)

S3 Table Comparison of differentially expressed proteins and associated values between CDC-treated and PBS-treated PAH rodent RV free wall myocardium. (XLSX)

S4 Table CDC-treated samples and their related gene ontologies. Shown here are selected, significantly changed proteins within the CDC-treated samples and their associated gene ontologies, related phenotypic pathways, and functions.
(DOCX)

S5 Table Significant upstream regulators obtained from differentially expressed proteins in CDC-treated samples compared to placebo control groups. DEPs are the number of differentially expressed proteins assigned to each upstream protein regulator. ND dictates an undefined number, whereas the rest of the values refer to the number of proteins associated with the particular phenotypic pathway and with the specific upstream regulator.
(DOCX)

S1 Fig The differential expressed proteins associated with autophagy. (A) Autophagy-associated proteins that are differentially expressed in PAH-PBS compared to PAH-CDC animals (cyan and orange bars, respectively) with direction indication either up or down regulation (B) Table listing the fold change for each protein of the relevant samples. Darker purple colors represent down-regulation and lighter pink colors represent up-regulation of proteins. Values denoted by * represent proteins that were identified only in certain samples (only in CDC-treated or only in control sample), therefore may not be indicative of true changes.
(DOCX)

S2 Fig Significant differential protein expression of mitochondrial proteins. (A) Mitochondrial-associated proteins that are differentially expressed in PAH-PBS compared to PAH-CDC animals (cyan and orange bars, respectively) with direction indication either up or down regulation (B) Table listing the fold change of each protein for the relevant samples. Darker purple colors represent down regulation and lighter pink colors represent up-regulation of proteins. Values denoted by * represent proteins that were identified only in certain samples (only in CDC-treated or only in control sample), therefore may not be indicative of true changes.
(DOCX)

S3 Fig Secondary antibody-only control images. Representative images for (A) anti-rabbit Alexa 488 secondary antibody-only staining and (B) anti-mouse Alexa 546 secondary antibody-only staining. Nuclear DAPI staining is shown in blue.
(DOCX)

S4 Fig Measurements of TAPSE in healthy, PAH-PBS and PAH-CDC animals across four weeks, post-hypoxia. TAPSE values expressed in millimeters (mm) for all animals in each treatment group across four weeks.
(DOCX)

## References

[1] Hendriks PM, Staal DP, van de Groep LD, van den Toorn LM, Chandoesing PP, Kauling RM, et al. The evolution of survival of pulmonary arterial hypertension over 15 years. Pulm Circ. 2022;12:e12137. doi: 10.1002/pul2.12137 36268054 PMC9579738

[2] van de Veerdonk MC, Bogaard HJ, Voelkel NF. The right ventricle and pulmonary hypertension. Heart Fail Rev. 2016;21(3):259–71. doi: 10.1007/s10741-016-9526-y 26833318

[3] Vonk-Noordegraaf A, Haddad F, Chin KM, Forfia PR, Kawut SM, Lumens J, et al. Right heart adaptation to pulmonary arterial hypertension: physiology and pathobiology. J Am Coll Cardiol. 2013;62:D22–33. doi: 10.1016/j.jacc.2013.10.027 24355638

[4] Voelkel NF, Gomez-Arroyo J, Abbate A, Bogaard HJ. Mechanisms of right heart failure-A work in progress and a plea for failure prevention. Pulm Circ. 2013;3:137–43. doi: 10.4103/2045-8932.109957 23662190 PMC3641721

[5] Spruijt OA, de Man FS, Groepenhoff H, Oosterveer F, Westerhof N, Vonk-Noordegraaf A, et al. The effects of exercise on right ventricular contractility and right ventricular-arterial coupling in pulmonary hypertension. Am J Respir Crit Care Med. 2015;191:1050–7. 25710636 10.1164/rccm.201412-2271OC

[6] Ryan JJ, Huston J, Kutty S, Hatton ND, Bowman L, Tian L, et al. Right ventricular adaptation and failure in pulmonary arterial hypertension. Can J Cardiol. 2015;31:391–406. 25840092 10.1016/j.cjca.2015.01.023 PMC4385216

[7] Piao L, Fang Y-H, Parikh KS, Ryan JJ, D’Souza KM, Theccanat T, et al. GRK2-mediated inhibition of adrenergic and dopaminergic signaling in right ventricular hypertrophy: therapeutic implications in pulmonary hypertension. Circulation. 2012;126:2859–69. doi: 10.1161/CIRCULATIONAHA.112.109868 23124027 PMC4459732

[8] Bogaard HJ, Natarajan R, Henderson SC, Long CS, Kraskauskas D, Smithson L, et al. Chronic pulmonary artery pressure elevation is insufficient to explain right heart failure. Circulation. 2009;120:1951–60. 19884466 10.1161/CIRCULATIONAHA.109.883843

[9] Gomez-Arroyo J, Sandoval J, Simon MA, Dominguez-Cano E, Voelkel NF, Bogaard HJ. Treatment for pulmonary arterial hypertension-associated right ventricular dysfunction. Ann Am Thorac Soc. 2014;11:1101–15. 25079379 10.1513/AnnalsATS.201312-425FR

[10] Reddy S, Bernstein D. Molecular mechanisms of right ventricular failure. Circulation. 2015;132(18):1734–42. doi: 10.1161/CIRCULATIONAHA.114.012975 26527692 PMC4635965

[11] Otsuki S, Sawada H, Yodoya N, Shinohara T, Kato T, Ohashi H, et al. Potential contribution of phenotypically modulated smooth muscle cells and related inflammation in the development of experimental obstructive pulmonary vasculopathy in rats. PLoS One. 2015;10:e0118655. doi: 10.1371/journal.pone.0118655 25714834 PMC4340876

[12] Campian ME, Hardziyenka M, de Bruin K, van Eck-Smit BL, de Bakker JM, Verberne HJ, et al. Early inflammatory response during the development of right ventricular heart failure in a rat model. Eur J Heart Fail. 2010;12:653–658. 20495202 10.1093/eurjhf/hfq066

[13] Sutendra G, Dromparis P, Paulin R, Zervopoulos S, Haromy A, Nagendran J, et al. A metabolic remodeling in right ventricular hypertrophy is associated with decreased angiogenesis and a transition from a compensated to a decompensated state in pulmonary hypertension. J Mol Med (Berl). 2013;91:1315–27. doi: 10.1007/s00109-013-1059-4 23846254

[14] Potus F, Ruffenach G, Dahou A, Thebault C, Breuils-Bonnet S, Tremblay E, et al. Downregulation of MicroRNA-126 contributes to the failing right ventricle in pulmonary arterial hypertension. Circulation. 2015;132:932–943. doi: 10.1161/CIRCULATIONAHA.115.016382 26162916

[15] Kümpers P, Nickel N, Lukasz A, Golpon H, Westerkamp V, Olsson KM, et al. Circulating angiopoietins in idiopathic pulmonary arterial hypertension. Eur Heart J. 2010;31:2291–300. doi: 10.1093/eurheartj/ehq226 20601390

[16] Zungu-Edmondson M, Shults NV, Wong CM, Suzuki YJ. Modulators of right ventricular apoptosis and contractility in a rat model of pulmonary hypertension. Cardiovasc Res. 2016;110:30–39. 26790474 10.1093/cvr/cvw014 PMC4798045

[17] Talati M, Hemnes A. Fatty acid metabolism in pulmonary arterial hypertension: role in right ventricular dysfunction and hypertrophy. Pulm Circ. 2015;5:269–278. doi: 10.1086/681227 26064451 PMC4449237

[18] Marbán E. The secret life of exosomes: What bees can teach us about next-generation therapeutics. J Am Coll Cardiol. 2018;71:193–200. doi: 10.1016/j.jacc.2017.11.013 29325643 PMC5769161

[19] Marban E. A mechanistic roadmap for the clinical application of cardiac cell therapies. Nat Biomed Eng. 2018;2:353–361. 30740264 10.1038/s41551-018-0216-z PMC6366940

[20] Ibrahim AG, Cheng K, Marbán E. Exosomes as critical agents of cardiac regeneration triggered by cell therapy. Stem Cell Rep. 2014;2:606–619. doi: 10.1016/j.stemcr.2014.04.006 24936449 PMC4050492

[21] Rogers RG, Ciullo A, Marbán E, Ibrahim AG. Extracellular vesicles as therapeutic agents for cardiac fibrosis. Front Physiol. 2020;11:479. doi: 10.3389/fphys.2020.00479 32528309 PMC7255103

[22] Smith RR, Barile L, Cho HC, Leppo MK, Hare JM, Messina E, et al. Regenerative potential of cardiosphere-derived cells expanded from percutaneous endomyocardial biopsy specimens. Circulation. 2007;115:896–908. doi: 10.1161/CIRCULATIONAHA.106.655209 17283259

[23] Davis DR, Zhang Y, Smith RR, Cheng K, Terrovitis J, Malliaras K, et al. Validation of the cardiosphere method to culture cardiac progenitor cells from myocardial tissue. PLoS One. 2009;4(9):e7195. doi: 10.1371/journal.pone.0007195 19779618 PMC2745677

[24] Chimenti I, Smith RR, Li T-S, Gerstenblith G, Messina E, Giacomello A, et al. Relative roles of direct regeneration versus paracrine effects of human cardiosphere-derived cells transplanted into infarcted mice. Circ Res. 2010;106:971–80. 20110532 10.1161/CIRCRESAHA.109.210682 PMC4317351

[25] Gallet R, de Couto G, Simsolo E, Valle J, Sun B, Liu W, et al. Cardiosphere-derived cells reverse heart failure with preserved ejection fraction (HFpEF) in rats by decreasing fibrosis and inflammation. JACC Basic Transl Sci. 2016;1:14–28. doi: 10.1016/j.jacbts.2016.01.003 27104217 PMC4834906

[26] Tseliou E, Reich H, de Couto G, Terrovitis J, Sun B, Liu W, et al. Cardiospheres reverse adverse remodeling in chronic rat myocardial infarction: Roles of soluble endoglin and Tgf-beta signaling. Basic Res Cardiol. 2014;109:443. 25245471 10.1007/s00395-014-0443-8

[27] Makkar RR, Smith RR, Cheng K, Malliaras K, Thomson LE, Berman D, et al. Intracoronary cardiosphere-derived cells for heart regeneration after myocardial infarction (CADUCEUS): a prospective, randomised phase 1 trial. Lancet. 2012;379:895–904. doi: 10.1016/S0140-6736(12)60195-0 22336189 PMC4326004

[28] Malliaras K, Makkar RR, Smith RR, Cheng K, Wu E, Bonow RO, et al. Intracoronary cardiosphere-derived cells after myocardial infarction: evidence of therapeutic regeneration in the final 1-year results of the CADUCEUS trial (CArdiosphere-Derived aUtologous stem CElls to reverse ventricUlar dySfunction). J Am Coll Cardiol. 2014;63:110–22. 24036024 10.1016/j.jacc.2013.08.724 PMC3947063

[29] Aminzadeh MA, Tseliou E, Sun B, Cheng K, Malliaras K, Makkar RR, et al. Therapeutic efficacy of cardiosphere-derived cells in a transgenic mouse model of non-ischaemic dilated cardiomyopathy. Eur Heart J. 2015;36:751–62. doi: 10.1093/eurheartj/ehu196 24866210 PMC4368856

[30] Cambier L, de Couto G, Ibrahim A, Echavez AK, Valle J, Liu W, et al. Y RNA fragment in extracellular vesicles confers cardioprotection via modulation of IL-10 expression and secretion. EMBO Mol Med. 2017;9:337–52. 28167565 10.15252/emmm.201606924 PMC5331234

[31] Middleton RC, Fournier M, Xu X, Marbán E, Lewis MI. Therapeutic benefits of intravenous cardiosphere-derived cell therapy in rats with pulmonary hypertension. PLoS One. 2017;12:e0183557. doi: 10.1371/journal.pone.0183557 28837618 PMC5570343

[32] Akhmerov A, Rogers R, de Couto G, Valle J, Li L, Ibrahim A, et al. Regulatory T cell activation, proliferation, and reprogramming induced by extracellular vesicles. J Heart Lung Transplant. 2021;40(11):1387–95. doi: 10.1016/j.healun.2021.06.005 34281778 PMC8570987

[33] de Couto G, Gallet R, Cambier L, Jaghatspanyan E, Makkar N, Dawkins JF, et al. Exosomal MicroRNA transfer into macrophages mediates cellular postconditioning. Circulation. 2017;136:200–14. doi: 10.1161/CIRCULATIONAHA.116.024590 28411247 PMC5505791

[34] Cheng M, Qin G. Progenitor cell mobilization and recruitment: SDF-1, CXCR4, α4-integrin, and c-kit. Prog Mol Biol Transl Sci. 2012;111:243–64. doi: 10.1016/B978-0-12-398459-3.00011-3 22917234 PMC3556394

[35] Oka M, Homma N, Taraseviciene-Stewart L, Morris KG, Kraskauskas D, Burns N, et al. Rho kinase-mediated vasoconstriction is important in severe occlusive pulmonary arterial hypertension in rats. Circ Res. 2007;100:923–9. 17332430 10.1161/01.RES.0000261658.12024.18

[36] Chaudhary KR, Deng Y, Yang A, Cober ND, Stewart DJ. Penetrance of severe pulmonary arterial hypertension in response to vascular endothelial growth factor receptor 2 blockade in a genetically prone rat model is reduced by female sex. J Am Heart Assoc. 2021;10(15):e019488. doi: 10.1161/JAHA.120.019488 34315227 PMC8475703

[37] Davis DR, Kizana E, Terrovitis J, Barth AS, Zhang Y, Smith RR, et al. Isolation and expansion of functionally-competent cardiac progenitor cells directly from heart biopsies. J Mol Cell Cardiol. 2010;49(2):312–21. doi: 10.1016/j.yjmcc.2010.02.019 20211627 PMC2885498

[38] de Raaf MA, Schalij I, Gomez-Arroyo J, Rol N, Happe C, de Man FS, et al. SuHx rat model: partly reversible pulmonary hypertension and progressive intima obstruction. Eur Respir J. 2014;44:160–8. 24791833 10.1183/09031936.00204813

[39] Kuang M, Chen Y, Xing Y, Du M, Feng H, Yang Q, et al. Echocardiographic evaluation of right heart failure which might be associated with DNA damage response in SU5416-hypoxia induced pulmonary hypertension rat model. Respir Res. 2023;24:202. 37592245 10.1186/s12931-023-02501-7 PMC10433698

[40] Nguyen QT, Nsaibia MJ, Sirois MG, Calderone A, Tardif JC, Fen Shi Y, et al. PBI-4050 reduces pulmonary hypertension, lung fibrosis, and right ventricular dysfunction in heart failure. Cardiovasc Res. 2020;116:171–82. 30753422 10.1093/cvr/cvz034

[41] Cheng K, Malliaras K, Li T-S, Sun B, Houde C, Galang G, et al. Magnetic enhancement of cell retention, engraftment, and functional benefit after intracoronary delivery of cardiac-derived stem cells in a rat model of ischemia/reperfusion. Cell Transplant. 2012;21(6):1121–35. doi: 10.3727/096368911X627381 22405128 PMC4323149

[42] Molina EJ, Palma J, Gupta D, Gaughan JP, Houser S, Macha M. Right ventricular effects of intracoronary delivery of mesenchymal stem cells (MSC) in an animal model of pressure overload heart failure. Biomed Pharmacother. 2009;63:767–72. 18993028 10.1016/j.biopha.2008.09.004

[43] Eng JK, Jahan TA, Hoopmann MR. Comet: an open-source MS/MS sequence database search tool. Proteomics. 2013;13:22–4. doi: 10.1002/pmic.201200439 23148064

[44] Craig R, Beavis RC. TANDEM: matching proteins with tandem mass spectra. Bioinformatics. 2004;20:1466–7. doi: 10.1093/bioinformatics/bth092 14976030

[45] Keller A, Nesvizhskii AI, Kolker E, Aebersold R. Empirical statistical model to estimate the accuracy of peptide identifications made by MS/MS and database search. Anal Chem. 2002;74:5383–92. 12403597 10.1021/ac025747h

[46] MacLean B, Tomazela DM, Shulman N, Chambers M, Finney GL, Frewen B, et al. Skyline: an open source document editor for creating and analyzing targeted proteomics experiments. Bioinformatics. 2010;26:966–8. doi: 10.1093/bioinformatics/btq054 20147306 PMC2844992

[47] Sundararaman N, Go J, Robinson AE, Mato JM, Lu SC, Van Eyk JE, Venkatraman V. PINE: An automation tool to extract and visualize protein-centric functional networks. J Am Soc Mass Spectrom. 2020;31:1410–21. doi: 10.1021/jasms.0c00032 32463229 PMC10362945

[48] Singh S, Chakravarty T, Chen P, Akhmerov A, Falk J, Friedman O, et al. Allogeneic cardiosphere-derived cells (CAP-1002) in critically ill COVID-19 patients: compassionate-use case series. Basic Res Cardiol. 2020;115(4):36. doi: 10.1007/s00395-020-0795-1 32399655 PMC7214858

[49] Rogers RG, Fournier M, Sanchez L, Ibrahim AG, Aminzadeh MA, Lewis MI, Marban E, et al. Disease-modifying bioactivity of intravenous cardiosphere-derived cells and exosomes in mdx mice. JCI Insight. 2019;4:e125754. doi: 10.1172/jci.insight.125754 30944252 PMC6483717

[50] de Couto G. Macrophages in cardiac repair: Environmental cues and therapeutic strategies. Exp Mol Med. 2019;51:1–10. doi: 10.1038/s12276-019-0269-4 31857583 PMC6923399

[51] Aminzadeh MA, Rogers RG, Fournier M, Tobin RE, Guan X, Childers MK, et al. Exosome-mediated benefits of cell therapy in mouse and human models of duchenne muscular dystrophy. Stem Cell Reports. 2018;10:942–55. doi: 10.1016/j.stemcr.2018.01.023 29478899 PMC5918344

[52] Kobayashi T, Kim JD, Naito A, Yanagisawa A, Jujo-Sanada T, Kasuya Y, et al. Multi-omics analysis of right ventricles in rat models of pulmonary arterial hypertension: Consideration of mitochondrial biogenesis by chrysin. Int J Mol Med. 2022;49. 10.3892/ijmm.2022.5124 PMC8989426 35315498

[53] Rain S, Handoko ML, Trip P, Gan CT-J, Westerhof N, Stienen GJ, et al. Right ventricular diastolic impairment in patients with pulmonary arterial hypertension. Circulation. 2013;128(18):2016–25, 1–10. doi: 10.1161/CIRCULATIONAHA.113.001873 24056688

[54] Schiaffino S, Rossi AC, Smerdu V, Leinwand LA, Reggiani C. Developmental myosins: expression patterns and functional significance. Skelet Muscle. 2015;5:22. doi: 10.1186/s13395-015-0046-6 26180627 PMC4502549

[55] Wang TY, Arking DE, Maleszewski JJ, Fox-Talbot K, Nieuwenhuis TO, Santhanam L, et al. Human cardiac myosin light chain 4 (MYL4) mosaic expression patterns vary by sex. Sci Rep. 2019;9:12681. doi: 10.1038/s41598-019-49191-0 31481666 PMC6722118

[56] Gupta N, Bhaskar AS, and Lakshmana Rao PV. Transcriptional regulation and activation of the mitogen-activated protein kinase pathway after Japanese encephalitis virus infection in neuroblastoma cells. FEMS Immunol Med Microbiol. 2011;62:110–21. 21320173 10.1111/j.1574-695X.2011.00792.x

[57] Kyriakis JM, Avruch J. Mammalian MAPK signal transduction pathways activated by stress and inflammation: a 10-year update. Physiol Rev. 2012;92:689–737. doi: 10.1152/physrev.00028.2011 22535895

[58] Liu T, Zhou Y, Ko KS, Yang H. Interactions between Myc and mediators of inflammation in chronic liver diseases. Mediators Inflamm. 2015:276850. doi: 10.1155/2015/276850 26508814 PMC4609837

[59] Zappasodi R, Ruggiero G, Guarnotta C, Tortoreto M, Tringali C, Cavane A, et al. HSPH1 inhibition downregulates Bcl-6 and c-Myc and hampers the growth of human aggressive B-cell non-Hodgkin lymphoma. Blood. 2015;125(11):1768–71. doi: 10.1182/blood-2014-07-590034 25573990

[60] Zhen Y, Liu Z, Yang H, Yu X, Wu Q, Hua S, et al. Tumor suppressor PDCD4 modulates miR-184-mediated direct suppression of C-MYC and BCL2 blocking cell growth and survival in nasopharyngeal carcinoma. Cell Death Dis. 2013;4(10):e872. doi: 10.1038/cddis.2013.376 24157866 PMC3824685

[61] Barst RJ, Chung L, Zamanian RT, Turner M, McGoon MD. Functional class improvement and 3-year survival outcomes in patients with pulmonary arterial hypertension in the REVEAL Registry. Chest. 2013;144:160–8. 23429998 10.1378/chest.12-2417

[62] Farber HW, Miller DP, Poms AD, Badesch DB, Frost AE, Muros-Le Rouzic E, et al. Five-Year outcomes of patients enrolled in the REVEAL Registry. Chest. 2015;148:1043–54. doi: 10.1378/chest.15-0300 26066077

[63] Humbert M, Sitbon O, Chaouat A, Bertocchi M, Habib G, Gressin V, et al. Survival in patients with idiopathic, familial, and anorexigen-associated pulmonary arterial hypertension in the modern management era. Circulation. 2010;122:156–63. 20585011 10.1161/CIRCULATIONAHA.109.911818

[64] van de Veerdonk MC, Marcus JT, Westerhof N, de Man FS, Boonstra A, Heymans MW, et al. Signs of right ventricular deterioration in clinically stable patients with pulmonary arterial hypertension. Chest. 2015;147:1063–71. 25376008 10.1378/chest.14-0701

[65] Rain S, Andersen S, Najafi A, Gammelgaard Schultz J, da Silva Goncalves Bos D, Handoko ML, et al. Ottenheijm CA, and de Man FS. Right ventricular myocardial stiffness in experimental pulmonary arterial hypertension: Relative contribution of fibrosis and myofibril stiffness. Circ Heart Fail. 2016;9. 10.1161/CIRCHEARTFAILURE.115.002636 PMC4956674 27370069

[66] van der Bruggen CEE, Tedford RJ, Handoko ML, van der Velden J, de Man FS. RV pressure overload: from hypertrophy to failure. Cardiovasc Res. 2017;113:1423–32. 28957530 10.1093/cvr/cvx145

[67] Qian C, Tio RA, Roks AJ, Boddeus KM, Harmsen MC, van Gilst WH, et al. A promising technique for transplantation of bone marrow-derived endothelial progenitor cells into rat heart. Cardiovasc Pathol. 2007;16:127–35. 17502241 10.1016/j.carpath.2006.11.008

[68] Tata A, Stoppel DC, Hong S, Ben-Zvi A, Xie T, Gu C. An image-based RNAi screen identifies SH3BP1 as a key effector of Semaphorin 3E-PlexinD1 signaling. J Cell Biol. 2014;205:573–90. doi: 10.1083/jcb.201309004 24841563 PMC4033773

[69] Tao Y, Hu K, Tan F, Zhang S, Zhou M, Luo J, et al. SH3-domain binding protein 1 in the tumor microenvironment promotes hepatocellular carcinoma metastasis through WAVE2 pathway. Oncotarget. 2016;7:18356–70. doi: 10.18632/oncotarget.7786 26933917 PMC4951293

[70] Rho SB, Song YJ, Lim MC, Lee S-H, Kim B-R, Park S-Y. Programmed cell death 6 (PDCD6) inhibits angiogenesis through PI3K/mTOR/p70S6K pathway by interacting of VEGFR-2. Cell Signal. 2012;24(1):131–9. doi: 10.1016/j.cellsig.2011.08.013 21893193

[71] Fitzgerald J. WARP: A unique extracellular matrix component of cartilage, muscle, and endothelial cell basement membranes. Anat Rec (Hoboken). 2020;303:1619–23. doi: 10.1002/ar.24087 30768857

[72] Deckx S, Carai P, Bateman J, Heymans S, Papageorgiou A-P. Breeding strategy determines rupture incidence in post-infarct healing WARPing cardiovascular research. PLoS One. 2015;10:e0139199. doi: 10.1371/journal.pone.0139199 26406320 PMC4583407

[73] Liang TW, Chiu HH, Gurney A, Sidle A, Tumas DB, Schow P, et al. Vascular endothelial-junctional adhesion molecule (VE-JAM)/JAM 2 interacts with T, NK, and dendritic cells through JAM 3. J Immunol. 2002;168:1618–26. doi: 10.4049/jimmunol.168.4.1618 11823489

[74] Tu J, Jin J, Chen X, Sun L, Cai Z. Altered cellular immunity and differentially expressed immune-related genes in patients with systemic sclerosis-associated pulmonary arterial hypertension. Front Immunol. 2022;13:868983. 35663995 10.3389/fimmu.2022.868983 PMC9159786

[75] Carneiro FRG, Lepelley A, Seeley JJ, Hayden MS, Ghosh S. An essential role for ECSIT in mitochondrial complex I assembly and mitophagy in macrophages. Cell Rep. 2018;22(10):2654–66. doi: 10.1016/j.celrep.2018.02.051 29514094 PMC5909989

[76] Xu L, Humphries F, Delagic N, Wang B, Holland A, Edgar KS, et al. ECSIT is a critical limiting factor for cardiac function. JCI Insight. 2021:6. 10.1172/jci.insight.142801 PMC8262467 34032637

[77] Stobdan T, Zhou D, Ao-Ieong E, Ortiz D, Ronen R, Hartley I, et al. Endothelin receptor B, a candidate gene from human studies at high altitude, improves cardiac tolerance to hypoxia in genetically engineered heterozygote mice. Proc Natl Acad Sci U S A. 2015;112:10425–30. 26240367 10.1073/pnas.1507486112 PMC4547246

[78] Morano M, Zacharzowski U, Maier M, Lange PE, Alexi-Meskishvili V, Haase H, et al. Regulation of human heart contractility by essential myosin light chain isoforms. J Clin Invest. 1996;98:467–73. doi: 10.1172/JCI118813 8755658 PMC507451

[79] Abdelaziz AI, Segaric J, Bartsch H, Petzhold D, Schlegel WP, Kott M, et al. Functional characterization of the human atrial essential myosin light chain (hALC-1) in a transgenic rat model. J Mol Med (Berl). 2004;82:265–74. 14985854 10.1007/s00109-004-0525-4

[80] Fewell JG, Hewett TE, Sanbe A, Klevitsky R, Hayes E, Warshaw D, et al. Functional significance of cardiac myosin essential light chain isoform switching in transgenic mice. J Clin Invest. 1998;101(12):2630–9. doi: 10.1172/JCI2825 9637696 PMC508853

[81] Liu Y, Wang S, Wang C, Song H, Han H, Hang P, et al. Upregulation of M(₃) muscarinic receptor inhibits cardiac hypertrophy induced by angiotensin II. J Transl Med. 2013;11:209. doi: 10.1186/1479-5876-11-209 24028210 PMC3819674

[82] Pham C, Munoz-Martin N, Lodder EM. The diverse roles of TNNI3K in cardiac disease and potential for treatment. Int J Mol Sci. 2021;22. 10.3390/ijms22126422 PMC8232738 34203974

[83] McDonald CM, Marban E, Hendrix S, Hogan N, Ruckdeschel Smith R, Eagle M, et al. Repeated intravenous cardiosphere-derived cell therapy in late-stage Duchenne muscular dystrophy (HOPE-2): a multicentre, randomised, double-blind, placebo-controlled, phase 2 trial. Lancet. 2022;399:1049–58. 35279258 10.1016/S0140-6736(22)00012-5

