# Supplementary material for: Impact of cardiosphere-derived cells on the maladapted right ventricular muscle in a rat sugen/hypoxia model of pulmonary hypertension with right ventricular dysfunction
Source: PLoS One. 2025 May 12;20(5):e0321895. doi: 10.1371/journal.pone.0321895 (PMC12068596; doi:10.1371/journal.pone.0321895)
Supplement: S4 Table — Shown here are selected, significantly changed proteins within the CDC-treated samples and their associated gene ontologies, related phenotypic pathways, and functions. (DOCX) [file pone.0321895.s004.docx]

| Representative CDC-specific protein changes linked to physiological changes | | | |
| --- | --- | --- | --- |
| Phenotype | Function | Protein (abbrev.) | Reference |
| Fibrosis | ECM | Collagen alpha-1 (II) chain (COL2A1ii) | ND |
| Fibrosis | Regulator of ECM | Rho family-interacting cell polarization regulator 2 (P4HA1) | ND |
| Fibrosis | Regulator of ECM | Sparc (SPARC) | ND |
| Fibrosis | Regulator of ECM | von Willebrand factor A domain-containing protein 1 (VWA1) | ND |
| Cardiomyocyte hypertrophy / Vascularity | Regulator of Ca^2+^ handling proteins and smooth muscle | Muscarinic acetylcholine receptor M3 (CHRM3) | PMID:10944224 |
| Cardiomyocyte hypertrophy | Regulator of Ca^2+^ handling proteins | Sarcoplasmic/endoplasmic reticulum calcium ATPase 3 (ATPA2A3) | PMID: 11956212 |
| Cardiomyocyte hypertrophy | Regulator of Ca^2+^ handling proteins | Voltage-dependent L-type calcium channel subunit alpha-1D (CACNA1D) | PMID: 21131953 |
| Cardiomyocyte hypertrophy | Regulator of Ca^2+^ handling proteins | Calcium/calmodulin-dependent protein kinase type II subunit gamma (CAMK2G) | PMID: 23283722 |
| Cardiomyocyte hypertrophy | Regulator of sarcomere contraction | Regulator of G-protein signaling 2 (RGS2) | PMID: 19127022 |
| Cardiomyocyte hypertrophy | Regulator of sarcomere contraction | Serine/Threonine-protein kinase (TNNI3K) | PMID: 24925317 |
| Cardiomyocyte hypertrophy | Regulator of sarcomere contraction | Src substrate cortactin (CTTN) | PMID: 24700464 |
| Cardiomyocyte hypertrophy | Cardiac muscle sarcomeric protein | Myosin light chain 4 (MYL4) | PMID: 16675844 |
| Vascularity / Fibrosis | ECM | Collagen alpha 1 (V) chain (COL5A1) | PMID: 16492673 |
| Vascularity | Regulator of smooth muscle and vascularity | Transcription factor HES-1 (HES1) | PMID: 20122914 |
| Vascularity | Regulator of smooth muscle and vascularity | Transmembrane emp24 domain-containing protein 2 (TMED2) | PMID:20178780 |
| Vascularity | Regulator of smooth muscle and vascularity | Phospholipid phosphatase 3 (PPAP2B) | PMID: 12925589 |
| Vascularity | Regulator of smooth muscle and vascularity | Junctional adhesion molecule C (JAM3) | PMID: 15994945 |
| Vascularity | Regulator of smooth muscle and vascularity | SH3 domain-binding protein 1 (SH3BP1) | PMID: 24841563 |
| Vascularity | Regulator of smooth muscle and vascularity | Programmed cell death 6 (PDCD6) | PMID: 21893193 |
| Vascularity | Regulator of vascular biology and ECM | Secreted protein acidic and rich in cysteine (SPARC) | PMID: 12867428 |
| Vascularity | Regulator of smooth muscle and angiogenesis | Synaptojanin 2-binding protein (SYNJ2BP) | PMID: 24025447 |
| Vascularity | Regulator of vascular biology | Epsin-1 (EPN1) | PMID: 28717225 |
| Vascularity | Smooth muscle protein | Calmodulin-1 (CAM) | PMID: 23040497 |
| Vascularity | Smooth muscle protein | Calponin-3 (CNN3) | PMID: 30518778 |
| Immune-associated response | Phagocytosis by macrophages | ATP-binding cassette sub-family A member 7 (ABCA7) | ND |
| Immune-associated response | Regulation of lymphocytes | Purine nucleoside phosphorylase (PNP) | ND |
| Immune-associated response | Regulation of T-cells | Rho family-interacting cell polarization regulator 2 (RIPOR2) | ND |
| Immune-associated response | Regulation of inflammation | Metalloreductase STEAP4 (STEAP4) | ND |
| Immune-associated response | Negative regulation of inflammation | Phospholipid phosphatase 3 (Plpp3) | ND |
| Immune-associated response | Regulation of inflammation and immune response | Likely ortholog of mouse signaling intermediate in Toll (ECSIT) | ND |
| Immune-associated response | Leukocyte Extravasation | Junctional adhesion molecule 3 (JAM3) | ND |
| Immune-associated response | Redox/iron control | Metalloreductase STEAP4 (STEAP4) | ND |

**S4 Table. CDC-treated samples and their related gene ontologies.** Shown here are selected, significantly changed proteins within the CDC-treated samples and their associated gene ontologies, related phenotypic pathways, and functions.
