# Supplementary material for: Impact of cardiosphere-derived cells on the maladapted right ventricular muscle in a rat sugen/hypoxia model of pulmonary hypertension with right ventricular dysfunction
Source: PLoS One. 2025 May 12;20(5):e0321895. doi: 10.1371/journal.pone.0321895 (PMC12068596; doi:10.1371/journal.pone.0321895)
Supplement: S5 Table — DEPs are the number of differentially expressed proteins assigned to each upstream protein regulator. ND dictates an undefined number, whereas the rest of the values refer to the number of proteins associated with the particular phenotypic pathway and with the specific upstream regulator. (DOCX) [file pone.0321895.s005.docx]

| **CDC-responsive differentially expressed proteins linked to a predictive upstream regulator *ND = Not Detected** | | | | | | | | |
| --- | --- | --- | --- | --- | --- | --- | --- | --- |
| **Primary Predicted Upstream regulator** | **Total # of DEPs** | **Angiogenesis** | **Immunoregulatory / Inflammatory** | **Protein Expression** | **Cell Proliferation** | **Metabolism** | **Protein Transport** | **Sarcomeric Regulation** |
| Myc proto-oncogene protein (MYC) | 43 | ND | 30% | 30% | ND | ND | ND | ND |
| Kirsten rat sarcoma virus (KRAS) | 26 | 17% | 17% | ND | 21% | ND | ND | ND |
| Cellular tumor antigen p53 (TP53) | 46 | ND | 15.2% | 19.6% | ND | 15.2% | ND | ND |
| Hepatocyte nuclear factor 4-alpha (NHF4A) | 55 | ND | 16.4% | 28.6% | 16.3% | ND | 24.5% | ND |
| Transforming growth factor beta-1 (TGFb-1) | 38 | ND | 26.3% | ND | 21.1% | ND | ND | 21.1% |

**S5 Table. Significant upstream regulators obtained from differentially expressed proteins in CDC-treated samples compared to placebo control groups.** DEPs are the number of differentially expressed proteins assigned to each upstream protein regulator. ND dictates an undefined number, whereas the rest of the values refer to the number of proteins associated with the particular phenotypic pathway and with the specific upstream regulator.
