# Supplementary material for: Impact of cardiosphere-derived cells on the maladapted right ventricular muscle in a rat sugen/hypoxia model of pulmonary hypertension with right ventricular dysfunction
Source: PLoS One. 2025 May 12;20(5):e0321895. doi: 10.1371/journal.pone.0321895 (PMC12068596; doi:10.1371/journal.pone.0321895)
Supplement: S2 Fig — (A) Mitochondrial-associated proteins that are differentially expressed in PAH-PBS compared to PAH-CDC animals (cyan and orange bars, respectively) with direction indication either up or down regulation (B) Table listing the fold change of each protein for the relevant samples. Darker purple colors represent down regulation and lighter pink colors represent up-regulation of proteins. Values denoted by * represent proteins that were identified only in certain samples (only in CDC-treated or only in control sample), therefore may not be indicative of true changes. (DOCX) [file pone.0321895.s007.docx]

**
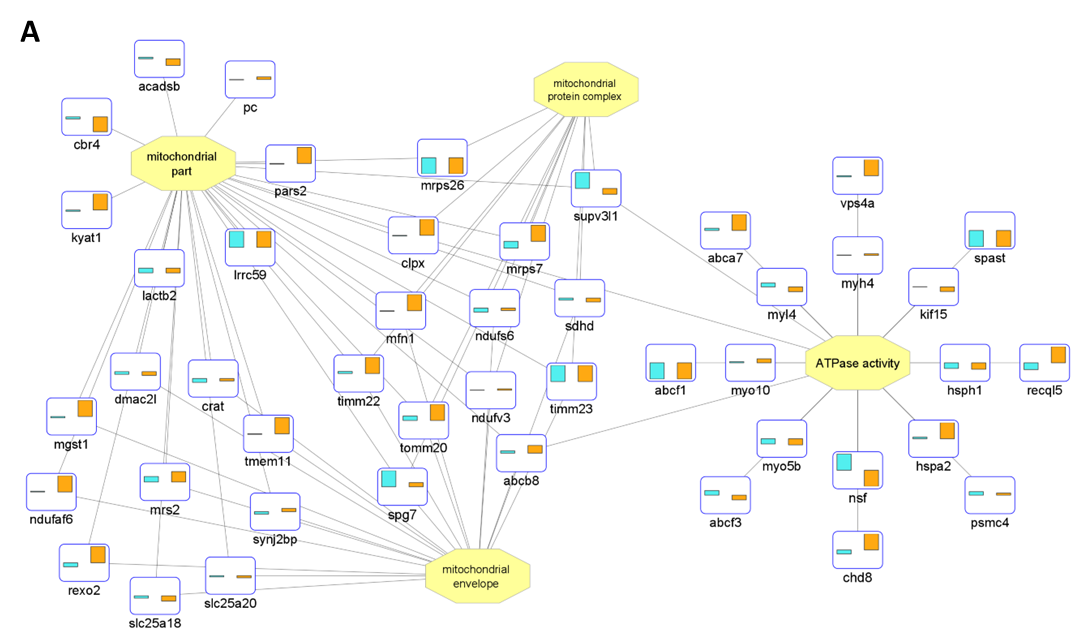
**

**
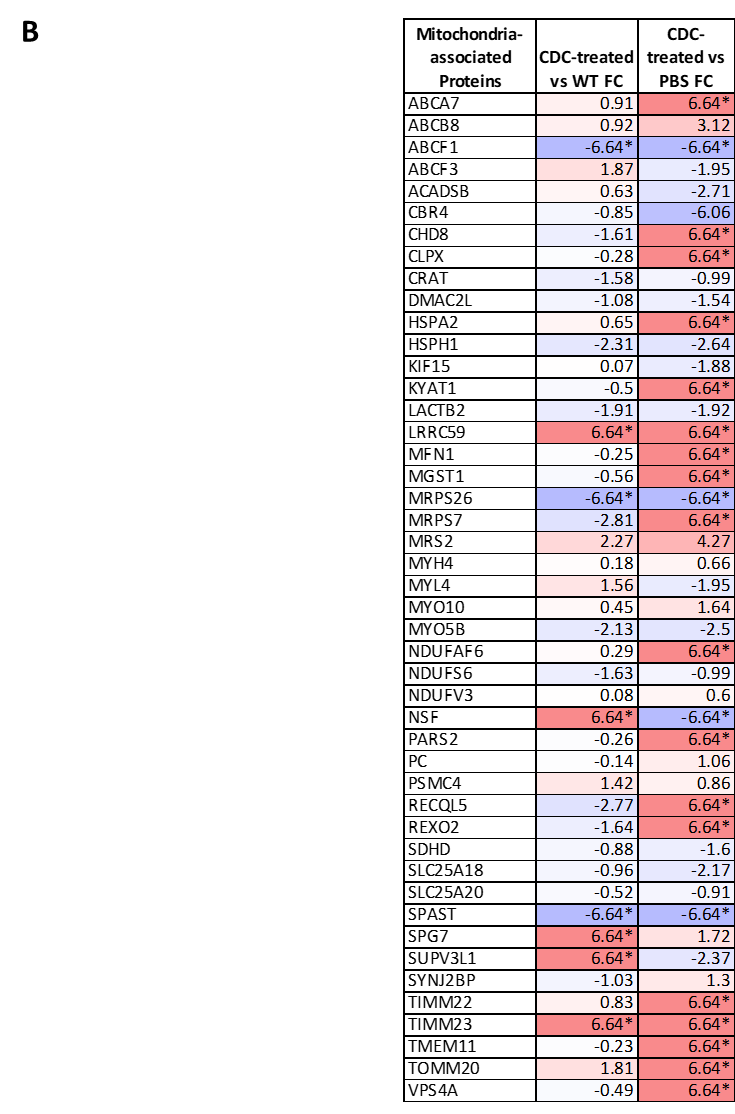
**

**S2 Fig. Significant differential protein expression of mitochondrial proteins. A.** Mitochondrial-associated proteins that are differentially expressed in PAH-PBS compared to PAH-CDC animals (cyan and orange bars, respectively) with direction indication either up or down regulation **B.** Table listing the fold change of each protein for the relevant samples. Darker purple colors represent down regulation and lighter pink colors represent up-regulation of proteins. Values denoted by ***** represent proteins that were identified only in certain samples (only in CDC-treated or only in control sample), therefore may not be indicative of true changes.
