# Supplementary material for: Impact of cardiosphere-derived cells on the maladapted right ventricular muscle in a rat sugen/hypoxia model of pulmonary hypertension with right ventricular dysfunction
Source: PLoS One. 2025 May 12;20(5):e0321895. doi: 10.1371/journal.pone.0321895 (PMC12068596; doi:10.1371/journal.pone.0321895)
Supplement: S3 Fig — Representative images for (A) anti-rabbit Alexa 488 secondary antibody-only staining and (B) anti-mouse Alexa 546 secondary antibody-only staining. Nuclear DAPI staining is shown in blue. (DOCX) [file pone.0321895.s008.docx]

**
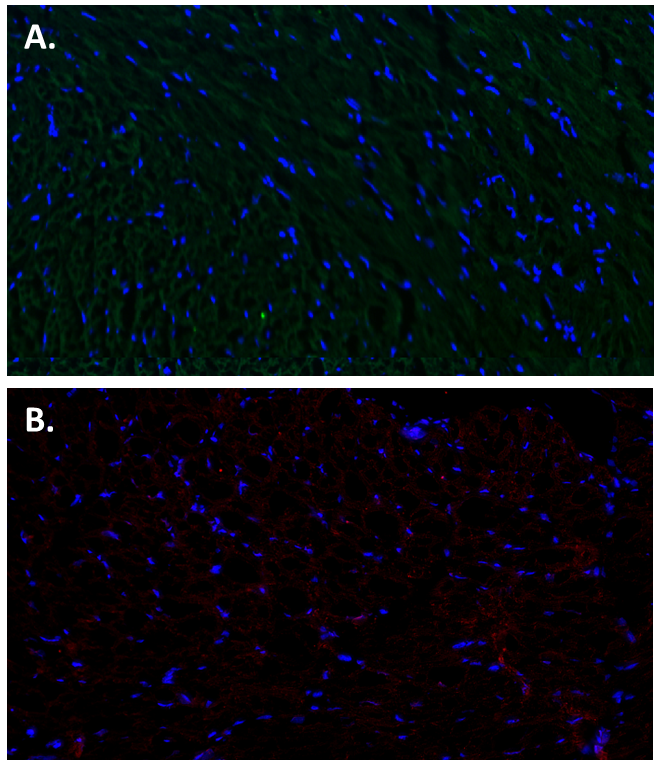
**

**S3 Fig. Secondary antibody-only control images.**

Representative images for **A.** anti-rabbit Alexa 488 secondary antibody-only staining and B. anti-mouse Alexa 546 secondary antibody-only staining. Nuclear DAPI staining is shown in blue.
