# Supplementary material for: Impact of cardiosphere-derived cells on the maladapted right ventricular muscle in a rat sugen/hypoxia model of pulmonary hypertension with right ventricular dysfunction
Source: PLoS One. 2025 May 12;20(5):e0321895. doi: 10.1371/journal.pone.0321895 (PMC12068596; doi:10.1371/journal.pone.0321895)
Supplement: S4 Fig — TAPSE values expressed in millimeters (mm) for all animals in each treatment group across four weeks. (DOCX) [file pone.0321895.s009.docx]

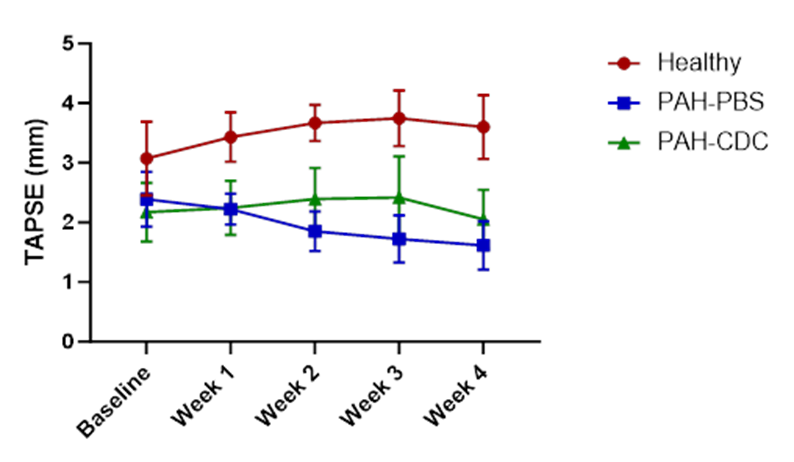


**S4 Fig. Measurements of TAPSE in healthy, PAH-PBS and PAH-CDC animals across four weeks, post-hypoxia.** TAPSE values expressed in millimeters (mm) for all animals in each treatment group across four weeks.
